# Diagnostic Accuracy of Web-Based COVID-19 Symptom Checkers: Comparison Study

**DOI:** 10.2196/21299

**Published:** 2020-10-06

**Authors:** Nicolas Munsch, Alistair Martin, Stefanie Gruarin, Jama Nateqi, Isselmou Abdarahmane, Rafael Weingartner-Ortner, Bernhard Knapp

**Affiliations:** 1 Data Science Department Symptoma Vienna Austria; 2 Medical Department Symptoma Attersee Austria; 3 Department of Internal Medicine Paracelsus Medical University Salzburg Austria

**Keywords:** COVID-19, symptom checkers, benchmark, digital health, symptom, chatbot, accuracy

## Abstract

**Background:**

A large number of web-based COVID-19 symptom checkers and chatbots have been developed; however, anecdotal evidence suggests that their conclusions are highly variable. To our knowledge, no study has evaluated the accuracy of COVID-19 symptom checkers in a statistically rigorous manner.

**Objective:**

The aim of this study is to evaluate and compare the diagnostic accuracies of web-based COVID-19 symptom checkers.

**Methods:**

We identified 10 web-based COVID-19 symptom checkers, all of which were included in the study. We evaluated the COVID-19 symptom checkers by assessing 50 COVID-19 case reports alongside 410 non–COVID-19 control cases. A bootstrapping method was used to counter the unbalanced sample sizes and obtain confidence intervals (CIs). Results are reported as sensitivity, specificity, F1 score, and Matthews correlation coefficient (MCC).

**Results:**

The classification task between COVID-19–positive and COVID-19–negative for “high risk” cases among the 460 test cases yielded (sorted by F1 score): Symptoma (F1=0.92, MCC=0.85), Infermedica (F1=0.80, MCC=0.61), US Centers for Disease Control and Prevention (CDC) (F1=0.71, MCC=0.30), Babylon (F1=0.70, MCC=0.29), Cleveland Clinic (F1=0.40, MCC=0.07), Providence (F1=0.40, MCC=0.05), Apple (F1=0.29, MCC=-0.10), Docyet (F1=0.27, MCC=0.29), Ada (F1=0.24, MCC=0.27) and Your.MD (F1=0.24, MCC=0.27). For “high risk” and “medium risk” combined the performance was: Symptoma (F1=0.91, MCC=0.83) Infermedica (F1=0.80, MCC=0.61), Cleveland Clinic (F1=0.76, MCC=0.47), Providence (F1=0.75, MCC=0.45), Your.MD (F1=0.72, MCC=0.33), CDC (F1=0.71, MCC=0.30), Babylon (F1=0.70, MCC=0.29), Apple (F1=0.70, MCC=0.25), Ada (F1=0.42, MCC=0.03), and Docyet (F1=0.27, MCC=0.29).

**Conclusions:**

We found that the number of correctly assessed COVID-19 and control cases varies considerably between symptom checkers, with different symptom checkers showing different strengths with respect to sensitivity and specificity. A good balance between sensitivity and specificity was only achieved by two symptom checkers.

## Introduction

In the modern world, large numbers of patients initially turn to various web-based sources for self-diagnoses of health concerns before seeking diagnoses from a trained medical professional. However, web-based sources have inherent problems, such as misinformation, misunderstandings, misleading advertisements, and varying quality [1]. Interactive web sources developed to provide web-based diagnoses are sometimes referred to as symptom checkers or chatbots [2,3]. Based on a list of entered symptoms and other factors, these symptom checkers return a list of potential diseases.

Web-based symptom checkers have become popular in the context of the novel COVID-19 pandemic, as access to physicians is reduced, concern in the population is high, and large amounts of misinformation are circulating the internet [1]. On COVID-19 symptom checker web pages, users are asked a series of COVID-19–specific questions; upon completion, an association between the answers and COVID-19 is given alongside behavioral recommendations, such as self-isolation.

In this context, COVID-19 symptom checkers are valuable tools for preassessment and screening during this pandemic; they can both ease pressure on clinicians and decrease footfall within hospitals. One example is practicing social distancing by not going to a physician’s waiting room when feeling sick. The importance of social distancing has been highlighted in the COVID-19 pandemic [4,5], the 2009 H1N1 influenza pandemic [6], and the 1918-1919 influenza pandemic [7] and is reviewed in [8]. Symptom checkers can also ease pressure on medical telephone hotlines [9,10] by reducing the number of human phone operators needed.

A large number of symptom checkers specific to COVID-19 have been developed. Empirical evidence (eg, a newspaper article [11]) suggests that their conclusions differ, with possible implications for the quality of the symptom assessment. To our knowledge, there are no studies comparing and evaluating COVID-19 symptom checkers.

In this paper, we present a study evaluating 10 different web-based COVID-19 symptom checkers using 50 COVID-19 cases extracted from the literature and 410 non–COVID-19 control cases of patients with other diseases. We found that the classifications of many patient cases by the COVID-19 symptom checkers differ. Therefore the accuracies of symptom checkers also differ.

## Methods

### COVID-19 Symptom Checkers

In April 2020, we conducted a Google search for COVID-19 symptom checkers using the search terms *COVID-19 symptom checker* and *Corona symptom checker*. All ten COVID-19 symptom checkers that we found and that were freely available on the internet between April 3 and 9, 2020, were included in this study (Table 1). Nine checkers were implemented in the English language, while one was in German. These symptom checkers were used in the versions available in this date range, and updates after this date were not considered for analysis.

As a baseline for the performance evaluation of the 10 web-based COVID-19 symptom checkers, we developed two additional simplistic symptom checkers. These two checkers evaluate and weigh the presence of COVID-19 symptom frequencies provided by the World Health Organization (WHO) [12] (see Multimedia Appendix 1) based on vector distance (SF-DIST) and cosine similarity (SF-COS). These approaches can be implemented in a few lines of code (see Multimedia Appendix 2).

**Table 1:** List of web-based COVID-19 symptom checkers included in this study.

Name	Reference
Ada	[13]
Apple	[14]
Babylon	[15]
CDC^a^	[16]
Cleveland Clinic	[17]
Docyet	[18]
Infermedica	[19]
Providence	[20]
Symptoma	[21]
Your.MD	[22]

^a^CDC: US Centers for Disease Control and Prevention.

### Clinical Cases

We used a total of 460 clinical cases to evaluate the performance of the COVID-19 symptom checkers. Each case lists both symptoms and the correct diagnosis alongside the age and sex of the patient when available. Details of the two case sets used are given below and in Table 2.

**Table 2:** Number of symptoms and age and sex distributions in each case set (N=460).

Characteristic		Case set		
			COVID-19, n=50	Control, n=410
**Number of symptoms**				
	Mean (SD)		8.4 (4.1)	9.8 (4.4)
	Median		7	9
**Age (years)**				
	Mean (SD)		45.6 (16.9)	38.6 (22.4)
	Median		45	38
**Sex, n (%)**				
	Male		25 (50)	238 (58)
	Female		21 (42)	160 (39)
	Unknown		4 (8)	12 (2.9)

#### COVID-19 Cases

A total of 50 COVID-19 cases were extracted by three trained physicians from the literature in March and April 2020 and are listed in Multimedia Appendix 3. Each case describes one patient’s medical situation (ie, symptoms experienced or COVID-19 contacts). The symptoms of each case were extracted separately from the COVID-19 engine construction and evaluation. The physicians entering the symptoms did not know how the engine would react to their symptom lists. To the best of our knowledge, we included all cases available at the time except for severe edge cases (eg, several severe comorbidities causing unrelated symptoms). Changes to the initial symptom lists were not allowed later.

#### Control Cases

The COVID-19 case data enabled us to evaluate the sensitivity of the symptom checkers. To evaluate the specificity, 410 control cases from the *British Medical Journal* (BMJ) were also sourced [23,24]. To allow a fair assessment, we only used cases containing at least one of the COVID-19 symptoms reported by the WHO [12] (see Multimedia Appendix 4). Classifying nonrelevant cases (eg, a fracture) would overestimate the symptom checkers’ specificity. Furthermore, these patients would not consult a web-based COVID-19 symptom checker. None of the 410 BMJ cases lists COVID-19 as the diagnosis, as the cases where collected before the COVID-19 outbreak.

### Mapping of Symptoms and Addressing Missing Inputs and Questions

Each of the symptom checkers has a different interface and different question sequences to reach the diagnosis. Therefore, we mapped the exhibited symptoms across our cases to the constrained input allowed by each checker via a synonym table and hierarchy created by a trained physician. For example, if a checker asked for “shortness of breath” but the case description listed “respiratory distress” or “(acute) dyspnea”, the symptom was still correctly used for this case and symptom checker.

Not all cases contained answers to all the questions of a checker. In such cases, the answer “I don't know” was chosen; if the “I don't know” answer option did not exist in a checker, “No” was used. In contrast, if a case contained information that did not fit any of the questions of the checker, this information was not used for this checker.

### Accuracy Evaluation

For statistical analysis, we used the following classification:

- True-positive : COVID-19 case classified as COVID-19
- False-positive: non–COVID-19 case classified as COVID-19
- True-negative: non–COVID-19 case classified as non–COVID-19
- False-negative: COVID-19 case classified as non–COVID-19

For each symptom checker, we calculated the following metrics:

Sensitivity (true-positive rate):


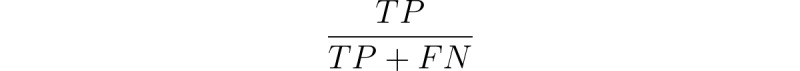


Specificity (true-negative rate):


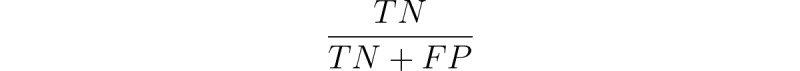


F1 score (harmonic mean of the precision and recall):


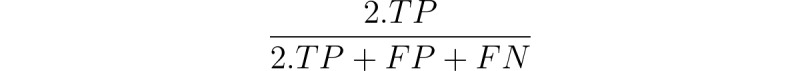


Matthews correlation coefficient (MCC):


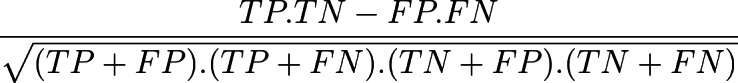


### Classification of the Outputs of the Symptom Checkers

Most COVID-19 symptom checkers return human-readable text that contains an association between the entered symptoms and COVID-19. We classified these associations into three different categories: high risk, medium risk, and low risk. Respective examples of a high, medium, and low risk classification are “There is a high risk that COVID-19 is causing your symptoms,” “Your symptoms are worrisome and may be related to COVID-19,” and “There's nothing at present to suggest that you have coronavirus (COVID-19). Please practice physical/social distancing.” Our full mapping of text outputs to risk for all symptom checkers and all text outputs is given in Multimedia Appendix 5.

Some symptom checkers only have two possible outputs: COVID-19 risk or no COVID-19 risk. To compare symptom checkers with three and two risk levels, we performed two different analyses: (1) medium risk and high risk were treated as COVID-19–positive (and low risk was treated as COVID-19 as negative), and (2) high risk was treated as COVID-19–positive (and low risk and medium risk were treated as COVID-19–negative).

### Bootstrapping

To evaluate the robustness of our statistical measures and account for the unbalanced dataset, we performed bootstrapping across our cases. A total of 3000 random samples consisting of 50 COVID-19 cases and 50 control cases was created by sampling with replacement from the original set of 50 COVID-19 cases and the 410 control cases.

## Results

**Figure 1:**
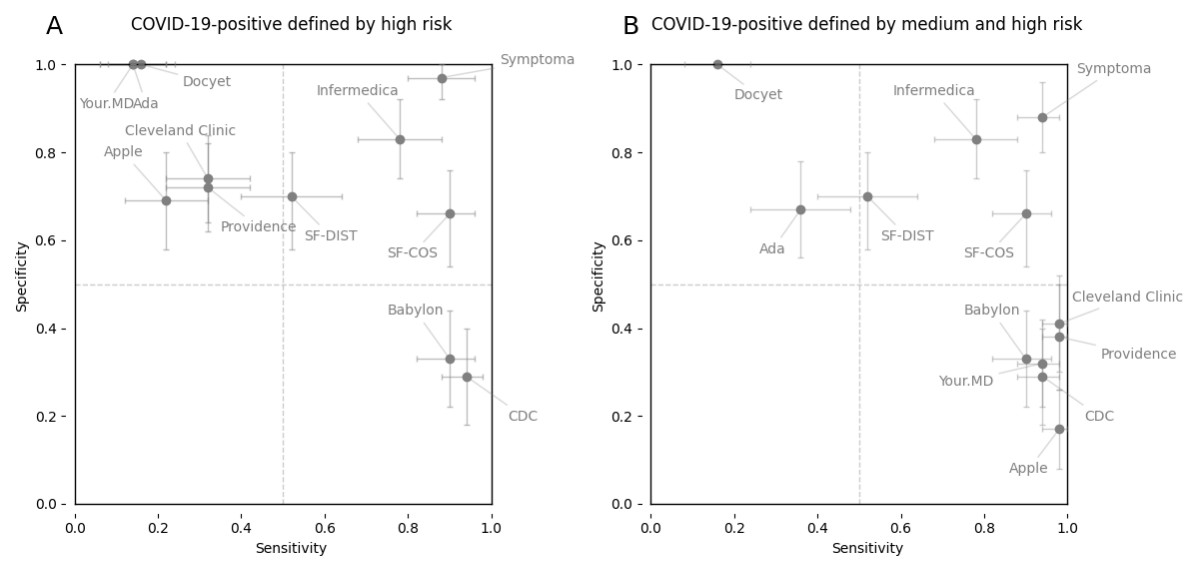
Sensitivities and specificities of web-based COVID-19 symptom checkers to COVID-19 cases and controls. The means of the 3000 random samples and 90% bootstrap CIs are reported as dots and crosses, respectively. (A) High risk: A COVID-19–positive prediction is defined only by a high risk result returned by a symptom checker. (B) Medium-high risk: A COVID-19–positive prediction is defined by either a medium risk or high risk result returned by a symptom checker. CDC: US Centers for Disease Control and Prevention; SF-COS: symptom frequency based on cosine similarity; SF-DIST: symptom frequency based on vector distance.

To analyze the performance of the 10 web-based symptom checkers, we calculated the sensitivity and the specificity of each symptom checker based on the cases described in the method section. Scatterplots of the sensitivity and specificity to COVID-19 of the different symptom checkers are given in Figure 1, and detailed numerical values are provided in Multimedia Appendix 6 and Multimedia Appendix 7. These symptom checkers fall approximately into four groups: upper left corner, lower right corner, central region, and upper right corner.

Further analysis of the true and false case classifications of these groups shows that the group in the upper left corner is composed of symptom checkers that require the presence of one (or few) highly specific symptoms to classify a case as COVID-19–positive (eg, “intensive contact with a COVID-19–positive person”). In this way, these symptom checkers miss many patients who are positive for COVID-19 who did not exactly report this highly specific symptom. In contrast, these highly specific symptoms are rarely present in non–COVID-19 cases. This results in low sensitivity and high specificity.

The group in the lower right corner is composed of symptom checkers that predict a case as COVID-19–positive based on the presence of one or few COVID-19 associated symptoms (eg, the presence of fever or cough is sufficient to predict a patient to be COVID-19–positive). These checkers classify almost every patient that has a respiratory disorder or viral infection as COVID-19–positive. As such, they do not miss many patients with COVID-19 but wrongly predict many patients who do not have COVID-19 to be COVID-19–positive. This results in low specificity and high sensitivity.

The group in the more central region is composed of symptom checkers that use a more balanced prediction but exhibit limited success in correctly classifying patients with and without COVID-19.

The group in the upper right corner is composed of symptom checkers that also use a more balanced model to associate symptoms to COVID-19; however, in this case, the classification of patients with and without COVID-19 is more successful. 

## Discussion

### Principal Findings

We classified 50 COVID-19 case descriptions from the recent literature as well as 410 non–COVID-19 control cases using 10 different web-based COVID-19 symptom checkers. Only 2/10 symptom checkers showed a reasonably good balance between sensitivity and specificity (Figure 1). Most other checkers were either too sensitive, classifying almost all patients as COVID-19–positive, or too specific, classifying many patients with COVID-19 as COVID-19–negative (Figure 1). For example, our BMJ control cases included a patient suffering from a pulmonary disease who presented with various symptoms, including fever, cough, and shortness of breath, which are the three most frequent symptoms associated with COVID-19. Additional symptoms and risk factors were not considered by most checkers. Namely, loss of appetite, green sputum, and a history of smoking can be used to discern a correct diagnosis of COVID-19–negative.

Furthermore, in terms of F1 score, most of the symptom checkers were outperformed by a simplistic symptom frequency vector approach; the F1 scores were 0.57 and 0.79 for SF-DIST and SF-COS, respectively. Notably, the cosine version showed surprisingly good results, outperforming 8/10 symptom checkers based on the F1 score.

In contrast, it could also be argued that sensitivity is more important for a COVID-19 symptom checker than specificity (ie, numerous false-positive COVID-19 diagnoses are not of concern as long as no COVID-19 infections are missed). However, it is not difficult to create a symptom checker that is 100% sensitive by simply returning every test as COVID-19–positive. While no checker does this 100% of the time, some checkers tend to declare every person who reports any flu-like symptom to be COVID-19–positive. This assesses every patient with allergic asthma (“shortness of breath”), heatstroke (“fever”), or heavy smoker (“cough”) to be COVID-19–positive. Therefore, we believe that a healthy balance between sensitivity and specificity is necessary for a useful checker. However, from the figure in this paper, readers can decide for themselves which balance between sensitivity and specificity is most useful and select the corresponding checker.

An additional aspect is that the developers of the 10 checkers may have had different purposes in mind during development. For example, they may have envisioned the checker to be a self-triage and recommendation tool or a likelihood predictor (as classified in [2]). In our study, we found that most checkers provide a certain likelihood as well as recommendations; therefore, classification is difficult. Therefore, we did not further subgroup the checkers in our analysis.

To our knowledge, this is the first scientific evaluation of web-based COVID-19 symptom checkers; however, there are a number of related studies evaluating symptom checkers. These include a study that evaluated 23 general-purpose symptom checkers based on 45 clinical case descriptions across a wide range of medical conditions and found that the correct diagnosis was listed among the top 20 results of the checkers in 58% of all cases on average [2]. The aforementioned study design was extended to five additional symptom checkers using ear, nose, and throat (ENT) cases, showing similar results [25]. Other evaluations include a study of symptom checkers used for knee pain cases; based on 527 patients and 26 knee problems, it was found that the physician’s diagnosis was present within the prediction list in 89% of the cases, while the specificity was only 27% [26]. In another study, an analysis of an automated self-assessment triage system for university students prior to an in-person consultation with a physician found that the system’s urgency rating agreed perfectly in only 39% of cases; meanwhile, for the remaining cases, the system tended to be more risk-averse than the physician [27]. Also, the applicability of web-based symptom checkers for 79 persons aged ≥50 years based on “think-aloud” protocols [28], deep learning algorithms for medical imaging [29], and services for urgent care [3] were evaluated.

The acceptability of the performance of a web-based symptom checker depends on the perspective and use of the results. In the case of COVID-19, a web-based assessment cannot fully replace a polymerase chain reaction (PCR) test, as some people are asymptomatic while others presenting with very specific COVID-19 symptoms may in fact have a very similar but different disease. Regardless, web-based COVID-19 symptom checkers can act as a first triage shield to avoid in-person physician visits or ease pressure on hospitals. Symptom checkers could even replace telephone triage lines in which non–medically trained personnel read a predefined sequence of questions. Although this was not part of this study, the authors believe that COVID-19 symptom checkers (if appropriately maintained and tested) may also be more reliable than the direct use of search engines such as Google or information via social media.

### Strengths and Limitations

The strength of this study lies in the fact that it is based on a large number of real patients’ case descriptions from the literature (n=460) and a detailed evaluation of the best performing symptom checker in terms of F1 score (Multimedia Appendix 8). In contrast, a potential weakness of this study lies in its use of real literature-based cases, which may have biased the test set to rather severe cases of COVID-19 because mild and uninteresting cases are usually not found in the literature. We countered this bias by not including extreme edge cases from the literature in our 50 COVID-19 cases. A limitation of our study is that the benchmarking represents a specific point in time (April 2020; see Methods) and underlying algorithms may change. However, this temporal limitation is present in all benchmarking studies as knowledge increases and software is updated. Another bias may be that our control case descriptions do not report a COVID-19 contact, even though, for example, a person with a cold may have had a COVID-19 contact (and did not become infected). Another limitation of this study is the nonstraightforward mapping of the symptom checker outputs to risk levels (Multimedia Appendix 5). The interpretation of the textual output is debatable in some cases. We countered this by allowing three different risk levels and merging them in two different ways (see Figure 1A and Figure 1B). Also, every symptom checker output was classified by multiple persons until consensus was reached.

### Conclusion

Symptom checkers are being widely used in response to the global COVID-19 pandemic. As such, quality assessment of these tools is critical. We show that various web-based COVID-19 symptom checkers vary widely in their predictive capabilities, with some performing equivalently to random guessing while others show strength in sensitivity, specificity, or both.

## Appendix

Multimedia Appendix 1 Symptom frequencies used in Multimedia Appendix 2.

Multimedia Appendix 2 Pseudocode of symptom frequencies based on vector distance (SF-DIST) and cosine similarity (SF-COS).

Multimedia Appendix 3 List of the COVID-19 cases.

Multimedia Appendix 4 List of COVID-19 symptoms according to the World Health Organization.

Multimedia Appendix 5 Mappings between output texts and risk levels of the symptom checkers. All mappings were independently performed by two different persons, and conflicts were resolved by a third person’s opinion.

Multimedia Appendix 6 Full table of sensitivities, specificities, accuracies, F1 scores, and Matthews correlation coefficients for all symptom checkers (COVID-19–positive was defined by “high risk” for nonbinary symptom checkers).

Multimedia Appendix 7 Full table of sensitivities, specificities, accuracies, F1 scores, and Matthews correlation coefficients for all symptom checkers (COVID-19–positive was defined by “medium risk or “high risk” for nonbinary symptom checkers).

Multimedia Appendix 8 Constraining symptoms for Symptoma.

Multimedia Appendix 9 Sensitivity vs specificity for all symptom checkers and Symptoma input constraint respectively by each symptom checker.

Multimedia Appendix 10 Full table of sensitivities, specificities, accuracies, F1 scores, and Matthews correlation coefficients for Symptoma constrained by each symptom checker (COVID-19–positive was defined by “high risk” for nonbinary symptom checkers).

Multimedia Appendix 11 Full table of sensitivities, specificities, accuracies, F1 scores, and Matthews correlation coefficients for Symptoma constrained by each symptom checker (COVID-19–positive was defined by “medium risk” or “high risk” for nonbinary symptom checkers).

Multimedia Appendix 12 Pairwise comparisons between all symptom checkers and Symptoma based on the MCC only if the subset of symptoms used by one checker is also used for Symptoma.

## Abbreviations

BMJ: British Medical Journal
ENT: ear, nose, and throat
MCC: Matthews correlation coefficient
SF-COS: symptom frequency based on cosine similarity
SF-DIST: symptom frequency based on vector distance
WHO: World Health Organization
