# Supplementary material for: Diagnostic Accuracy of Web-Based COVID-19 Symptom Checkers: Comparison Study
Source: J Med Internet Res. 2020 Oct 6;22(10):e21299. doi: 10.2196/21299 (PMC7541039; doi:10.2196/21299)
Supplement: Multimedia Appendix 1 [file jmir_v22i10e21299_app1.pdf]

Multimedia Appendix 1. Symptom frequencies used in Multimedia Appendix 2.

	COVID-19	Common cold	Influenza	Hay fever
Fever	87.9 [1]	15 [3]	68 [6]	NR
Fatigue	38.1 [2]	42 [4]	94 [6]	NR
Dry cough	67.7 [1]	80 [3]	93 [6]	22 [10]
Sneezing	NR	74 [4]	58 [7]	96 [11]
Malaise	14.8 [1]	30 [4]	94 [6]	NR
Rhinorrhea	4 [2]	95 [3]	91 [6]	62.1 [12]
Sore throat	13.9 [1]	70 [3]	84 [6]	30 [10]
Diarrhea	3.7 [1]	11 [4]	14.4 [8]	NR
Headache	13.6 [1]	80 [5]	91 [6]	50 [13]
Dyspnea	18.6 [1]	21 [4]	63 [9]	NR

[1] https://www.who.int/docs/default-source/coronaviruse/who-china-joint-mission-on-covid-19-final-report.pdf&sa=D&ust=1585147724205000&usg=AFQjCNHMTMLZYuYavNS7iXN_D8AaJvOYiw

[2] https://www.thelancet.com/action/showPdf?pii=S0140-6736%2820%2930211-7

[3] https://www.sciencedirect.com/science/article/pii/S0095454305703559?via%3Dihub

[4] https://www.ncbi.nlm.nih.gov/pubmed/3057962

[5] https://www.ncbi.nlm.nih.gov/pmc/articles/PMC4347877/pdf/nihms658637.pdf

[6] https://jamanetwork.com/journals/jamainternalmedicine/fullarticle/485554

[7] https://www.ncbi.nlm.nih.gov/pmc/articles/PMC4915903/

[8] https://www.ncbi.nlm.nih.gov/pmc/articles/PMC4676820/

[9] https://www.ncbi.nlm.nih.gov/pmc/articles/PMC3650195/

[10] https://www.ncbi.nlm.nih.gov/pubmed/10971479

[11] https://www.ncbi.nlm.nih.gov/pmc/articles/PMC5806744/

[12] https://www.researchgate.net/publication/307953143_Inverse_correlation_of_soluble_programmed_cell_death-1_ligand-1_sPD-L1_with_eosinophil_count_and_clinical_severity_in_allergic_rhinitis_patients

[13] https://www.ncbi.nlm.nih.gov/pubmed/17300360
