# Supplementary material for: Diagnostic Accuracy of Web-Based COVID-19 Symptom Checkers: Comparison Study
Source: J Med Internet Res. 2020 Oct 6;22(10):e21299. doi: 10.2196/21299 (PMC7541039; doi:10.2196/21299)
Supplement: Multimedia Appendix 2 [file jmir_v22i10e21299_app2.pdf]

Multimedia Appendix 2. Pseudo code of symptom frequencies based on vector distance (SF-DIST) and cosine similarity (SF-COS)

```
SF-COS :

Input : patient_symptoms (10x1), symptoms_frequencies (10x4)

if all patient_symptoms == 0:
       Return LOW RISK

For each column of symptoms_frequencies:
       similarity = cosine_similarity(patient_symptoms, symptoms_frequencies[column])

similarity = normalise(similarity)

similarity[COVID_19] = similarity[COVID_19]*(Area_Risk_Factor + Contact_Risk_Factor)

Output : return disease with maximum similarity
```

```
SF-DIST :

Input : patient_symptoms (10x1), symptoms_frequencies (10x4)

if all patient_symptoms == 0:
       Return LOW RISK

For each column of symptoms_frequencies:
       similarity = sum(abs((patient_symptoms - symptoms_frequencies[column]))

similarity = normalise(similarity)

similarity[COVID_19] = similarity[COVID_19]*(Area_Risk_Factor + Contact_Risk_Factor)

Output : return disease with maximum similarity
```
