# Supplementary material for: Diagnostic Accuracy of Web-Based COVID-19 Symptom Checkers: Comparison Study
Source: J Med Internet Res. 2020 Oct 6;22(10):e21299. doi: 10.2196/21299 (PMC7541039; doi:10.2196/21299)
Supplement: Multimedia Appendix 3 [file jmir_v22i10e21299_app3.pdf]

Multimedia Appendix 3. List of the COVID-19 cases

case	assessor	age	sex	symptoms	Source
1	1	50	FEMALE	Fever, Diarrhea, Anorexia, Asthenia, Dry Cough, Myalgia, China	1
1	2	50	FEMALE	Stay in COVID-19 Risk Area, Wuhan, fever, diarrhea, asthenia, anorexia, dry cough, myalgia, C-reactive protein increased, ground glass opacity (CT)	1
1	3	50	FEMALE	fever, diarrhea , anorexia, asthenia, Covid-19 risk area, dry cough, muscle ache, increased CRP , ground glass opacities, consolidation	1
2	1	10	FEMALE	Contact COVID-19 Case, Fever, Sputum Production, consolidations, Ground Glass Opacities (CT)	2
2	2	10	FEMALE	contact Covid-19 patient , fever, sputum production , consolidations, ground glass opacities	2
2	3	10	FEMALE	Contact COVID-19 Case, fever, sputum production, patchy consolidation, ground glass opacity	2
3	1	33	FEMALE	Wuhan, Fever, Cough, coarse breath sounds , Leukopenia, C-Reactive Protein Increased, Elevated Sedimentation Rate, D-Dimer Abnormal, Ground Glass Opacities (CT)	3
3	2	33	FEMALE	fever, cough, Stay in COVID-19 Risk Area, Wuhan, leukopenia, coarse breath sounds, elevated C-reactive protein, Elevated Sedimentation Rate, D-Dimer Abnormal, peripheral ground-glass opacitiy on CT	3
3	3	33	FEMALE	fever, cough, Wuhan, coarse breath sounds, leucopenia, increased CRP, increased erythrocyte sedimentation rate, increased D-dimer, ground glass opacities	3
4	1	41	FEMALE	Wuhan, Fever, Ground Glass Opacities (CT)	4
4	2	41	FEMALE	Wuhan, Stay in COVID-19 Risk Area, fever, ground-glass opacity on CT	4
4	3	41	FEMALE	Wuhan, fever, ground glass opacities	4
5	1	32	MALE	Wuhan, Fever, coarse breath sounds, Interleukin-6 Increased, right lower lobe consolidation, Bronchiectasis	5
5	2	32	MALE	Stay in COVID-19 Risk Area, Wuhan, cough, fever, IL-6 Increased, subpleural right lower lobe consolidation	5
5	3	32	MALE	Wuhan, cough, fever, coarse breath sounds, increased interleukin 6 , bronchiectasis, subpleural right lower lobe consolidation	5
6	1	45	FEMALE	Wuhan, Fever, Cough, coarse breath sounds, Neutrophil Count Increased, Lymphocytopenia, Elevated Sedimentation Rate, Interleukin-6 Increased, peripheral consolidation, Pneumonia, crazy-paving pattern	5
6	2	45	FEMALE	Wuhan, Stay in COVID-19 Risk Area, cough, fever, Neutrophil Count Increased, lymphopenia, Elevated Sedimentation Rate, IL-6 increased, bilateral peripheral consolidation	5
6	3	45	FEMALE	Wuhan, cough, fever, coarse breath sounds, increased neutrophil ratio , decreased lymphocyte , increased erythrocyte sedimentation rate, increased interleukin 6 , consolidation, interlobular septal thickening, crazy paving appearance, bronchiectasis	5

7	1	42	MALE	High Fever, Cough, Fatigue, Bilateral coarse breath sounds, Wet Rales, Leukopenia, Lymphocytes Increased, C-Reactive Protein Increased, Elevated Sedimentation Rate, Amyloid A Protein Increased, Aspartate Transaminase Increased, Alanine Aminotransferase Increased, Ground Glass Appearance, Ground Glass Opacities (CT), consolidation	6
7	2	42	MALE	Wuhan, Stay in COVID-19 Risk Area, high fever, cough, fatigue, bilateral coarse breath sounds, wet rales, leukopenia, lymphocytosis, Elevated Sedimentation Rate, C-Reactive Protein Increased, Aspartate Aminotransferase Increased, Alanine Aminotransferase Increased	6
7	3	42	MALE	Wuhan, fever, cough, fatigue , coarse breath sounds, wet rales , leukopenia, lymphocytosis, increased CRP, increased erythrocyte sedimentation rate, increased serum amyloid A protein, increased aspartate aminotransferase, increased alanine aminotransferase , ground glass opacities, ground glass consolidation	6
8	1	63	FEMALE	Wuhan, Fever, Cough, Dizziness, Constipation, Tachypnea, Rhonchi, Bilateral pneumonia	7
8	2	63	FEMALE	Wuhan, Stay in COVID-19 Risk Area, fever, cough, dizziness, constipation, Hyperpnea	7
8	3	63	FEMALE	Wuhan, fever , cough, dizziness, constipation, tachypnea , Rhonchi, pneumonia	7
9	1	63	MALE	Contact COVID-19 Case, Fever, Cough, Tachypnea, Rhonchi , Unilateral pneumonia	7
9	2	63	MALE	Contact COVID-19 Case, fever, cough, hyperpnea, Abnormal Chest CT Scan	7
9	3	63	MALE	contact Covid-19 patient , fever, cough, tachypnea, Rhonchi, pneumonia	7
10	1	19	MALE	Wuhan, Fever, Cough, Fatigue, Nasal Congestion, Rhinorrhea, Unilateral pneumonia	7
10	2	19	MALE	Wuhan, Stay in COVID-19 Risk Area, fever, cough, fatigue, nasal congestion, rhinorrhea, Abnormal CT scan	7
10	3	19	MALE	fever , cough, fatigue , Nasal congestion, Rhinorrhea, pneumonia , COVID-19 Risk Area	7
11	1	30	FEMALE	Fever, Chills, Fatigue, Cough, China	8
11	2	30	FEMALE	Wuhan, Stay in COVID-19 Risk Area, Contact COVID-19 Case, fever, chills, fatigue, cough	8
11	3	30	FEMALE	Covid-19 risk area, contact Covid-19 patient , fever, chills , fatigue, cough	8
12	1	31	MALE	Wuhan, Fever, Chills, Fatigue, Conjunctivitis, Cough	8
12	2	31	MALE	Wuhan, Stay in COVID-19 Risk Area, fever, chills, fatigue, conjunctivitis	8
12	3	31	MALE	fever, chills , fatigue, conjunctivitis, cough, Covid-19 risk area	8
13	1	48	MALE	Wuhan , Fever, Headache, Cough	9
13	2	48	MALE	Wuhan, Stay in COVID-19 Risk Area, fever, headache, cough	9

13	3	48	MALE	Covid-19 risk area, fever, headache , cough	9
14	1	55	MALE	Wuhan, Sore Throat, Myalgia, prominent bronchovascular bundle, Fever, Lymphocytopenia, C-Reactive Protein Increased, consolidation of both lower lobes, Ground Glass Opacities (CT)	10
14	2	55	MALE	Wuhan, Stay in COVID-19 Risk Area, Contact COVID-19 Case, Fever, sore throat, bilateral patchy consolidation, myalgia	10
14	3	55	MALE	Covid-19 risk area , sore throat, myalgia, prominent bronchovascular bundle, fever, lymphopenia, increased CRP, bilateral patch consolidation, ground glass opacities, interlobular septal thickening	10
15	1	50	MALE	Fever, Chills, Cough, Fatigue, Dyspnea, Wuhan, Leukopenia, Lymphocytopenia, multiple patchy shadows in both lungs, C-Reactive Protein Increased	11
15	2	50	MALE	fever, chills, cough, fatigue, dyspnea, Stay in COVID-19 Risk Area, patchy shadow on X-ray	11
15	3	50	MALE	fever, chills, cough, fatigue, shortness of breath, Covid-19 risk area , diffuse gridding shadow lung	11
16	1	46	FEMALE	Fever, Sore Throat, Cough, Chest Discomfort, Wuhan, Ground Glass Opacities (CT)	12
16	2	46	FEMALE	fever, sore throat, cough, chest distress, Stay in COVID-19 Risk Area, Wuhan, ground-glass opacity on CT	12
16	3	46	FEMALE	fever, sore throat , cough , chest distress, Covid-19 risk area , contact Covid-19 patient , ground glass opacities	12
17	1	34	FEMALE	Fever, history of hypothyroidism, Vaginal Bleeding, Abdominal Pain, Wuhan, Ground Glass Opacities (CT), Lymphocytopenia, Neutrophil Count Increased, C-Reactive Protein Increased	13
17	2	34	FEMALE	Wuhan, Stay in COVID-19 Risk Area, fever, ground-glass opacity on CT, lymphopenia, neutrophilia, C-reactive protein increased	13
17	3	34	FEMALE	Covid-19 risk area, vaginal bleeding, abdominal pain, fever, ground glass opacities, lymphopenia, neutrophilia, increased CRP	13
18	1	52	MALE	history of kidney transplantation, Immunosuppressive Therapy, Wuhan, Fatigue, Dyspnea, Dull Chest Pain, Chest Pain, Nausea, Loss of Appetite, Abdominal Pain, Dry Cough, Fever, Headache, Lymphocytopenia, Neutrophil Count Increased, Monocyte Count Increased, C-Reactive Protein Increased, Ground Glass Opacities (CT)	14
18	2	52	MALE	immunosuppression, Wuhan, Stay in COVID-19 Risk Area, fatigue, dyspnea, chest tightness, nausea, loss of appetite, abdominal pain, dry cough, fever, headache, lymphopenia, neutrophil count increased, C-reactive protein increased, multiple ground-glass opacity	14
18	3	52	MALE	chronic glomerulonephritis, immunosuppressive therapy, Covid-19 risk area , fatigue, dyspnea, tightness chest, chest pain, nausea, loss of appetite, abdominal pain, dry cough, fever, headache, decreased lymphocytes , increased CRP , ground glass shadow , increased neutrophil count	14
19	1	40	FEMALE	Fever, Dull Chest Pain, Fatigue, peripheral consolidations, ground-glass opacities in both lungs	15
19	2	40	FEMALE	fever, chest tightness, fatigue, ground glass opacity (CT), cough, leukopenia	15
19	3	40	FEMALE	fever, chest tightness, fatigue, glucose increased , ground glass opacities, peripheral consolidations, cough, decreased leukocytes, increased CRP	15

20	1	35	MALE	Wuhan, Fever, Cough, Neutrophil Count Increased, Lymphocytopenia, Glucose Increased, Ground Glass Appearance, C-Reactive Protein Increased	16
20	2	35	MALE	Stay in COVID-19 Risk Area, fever, cough, Wuhan, neutrophil count increased, lymphopenia, C-reactive protein increased, multiple patchy consolidations, ground-glass opacity (CT)	16
20	3	35	MALE	fever, cough, Covid-19 risk area, increased neutrophils, decreased lymphocytes , elevated glucose, increased CRP , consolidation, ground glass opacities	16
21	1	81	FEMALE	Abdominal Pain, Diarrhea, Sore Throat, Diamond Princess cruise ship	17
21	2	81	FEMALE	abdominal pain, sore throat , cruise ship, diarrhea , Japan	17
21	3	81	FEMALE	abdominal pain, watery diarrhea, sore throat, Diamond Princess	17
22	1	35	FEMALE	Fever, Chills, Myalgia, Wuhan, Nasal Congestion, Cough, Sputum Production	18
22	2	35	FEMALE	fever, chills, myalgia, Wuhan, Stay in COVID-19 Risk Area, cough, nasal congestion, sputum production	18
22	3	35	FEMALE	Covid-19 risk area , fever, chill, myalgia, nasal congestion, cough, sputum production	18
23	1	54	MALE	Fever, Dry Cough, ground-glass opacities in both lower lobes, China	19
23	2	54	MALE	Wuhan, Stay in COVID-19 Risk Area, hypertension, fever, dry cough, ground glass opacity (CT)	19
23	3	54	MALE	Covid-19 risk area, chills, muscle pain , hypertension, dry cough, consolidation, ground glass opacities	19
24	1	39	MALE	Fever, Sore Throat, Glucose Increased, Ground Glass Opacities (CT)	16
24	2	39	MALE	Contact COVID-19 Case, fever, ground-glass opacity (CT)	16
24	3	39	MALE	fever, soar throat , contact Covid-19 patient, , decreased aspartate aminotransferase, elevated glucose, ground glass opacities	16
25	1	39	MALE	Cough, frothy white sputum, Bilateral Pulmonary Opacities, Dyspnea, Decreased Oxygen Saturation, Altered Mental Status, Wuhan	20
25	2	39	MALE	Wuhan, Stay in COVID-19 Risk Area, fever, cough, sputum production, dyspnea, alteration of consciousness, patchy opacities (CT)	20
25	3	39	MALE	Covid-19 risk area, fever, cough , frothy white sputum, shortness of breath, patchy opacities, decreased mental state, decreased SpO2, ground glass opacities, increased albumin , increased Aspartate aminotransferase, increased Lactate dehydrogenase, Wuhan	20
26	1	21	FEMALE	Cough, Chills, Fever, frothy white sputum, Bilateral Pulmonary Opacities, Wuhan	21
26	2	21	FEMALE	Contact COVID-19 Case, fever, cough, chills, sputum production, leukopenia, thrombocytopenia	21
26	3	21	FEMALE	Covid-19 risk area, fever, cough, chills, frothy white sputum, patchy opacities, decreased lymphocytes , decreased platelet count	21

27	1	50	MALE	Diabetes Mellitus, Pneumonia, Respiratory Distress, Decreased Oxygen Saturation	22
27	2	50	MALE	diabetes mellitus, hyperlipidemia, respiratory distress	22
27	3	50	MALE	diabetes mellitus, penumonia, respiratory distress	22
28	1	47	FEMALE	Wuhan, Lethargy, Sore Throat, Dry Cough, Pleuritic Pain, Dyspnea, Fever, Bilateral Pulmonary Infiltrate, C-Reactive Protein Increased	23
28	2	47	FEMALE	Wuhan, lethargy, sore throat, dry cough, pleuritic chest pain, dyspnea, fever, tachycardia, C-reactive protein increased, bilateraly patchy infiltration	23
28	3	47	FEMALE	Covid-19 risk area, lethargy, sore throat, dry cough, pleuritic chest pain , dyspnea, fever, rhonchi, increased CRP, pulmonary infiltrate	23
29	1	32	MALE	Fever, Fatigue, Dizziness, Constipation, Rhonchi , Tachypnea, Bilateral pneumonia	7
29	2	32	MALE	fever, fatigue, dizziness, constipation, rhonchi on auscultation, hyperpnea, CT scan abnormal	7
29	3	32	MALE	Covid-19 risk area, fever, fatigue , dizziness , Constipation, Rhonchi, tachypnea , pneumonia	7
30	1	64	MALE	Fever, Dizziness, Headache, Malaise, Wuhan, bilateral multiple ground-glass opacities	24
30	2	64	MALE	fever, dizziness, headache, myalgia, Wuhan, Stay in COVID-19 Risk Area, ground-glass opacity	24
30	3	64	MALE	ground glass opacities, fever, dizziness, headache, aching limbs , Covid-19 risk area	24
31	1	79	FEMALE	history of coronary artery disease , Hypertension, Congestive Heart Failure, Syncope, Myalgia, Fever, Cough, Lymphocytopenia, Chills, Ground Glass Opacities (CT)	25
31	2	79	FEMALE	coronary artery disease, hypertension, myalgia, cough, fever, altered consciousness, lymphopenia, chills, ground-glass opacity (CT)	25
31	3	79	FEMALE	coronary artery disease , hypertension, myalgia, cough , fever, lymphopenia, chills, ground glass opacities	25
32	1	75	MALE	End Stage Renal Disease, Wuhan, Cough, Dull Chest Pain, Hypertension, Heart Failure, Chronic Obstructive Pulmonary Disease, pitting edema bilaterally, Ground Glass Opacities (CT), Decreased Oxygen Saturation	26
32	2	75	MALE	Wuhan, Stay in COVID-19 Risk Area, end-stage kidney disease, cough, chest tightness, hypertension, Chronic obstructive pulmonary disease, hyperpnea	26
32	3	75	MALE	Covid-19 risk area, cough , chest tightness, hypertension, chronic heart failure, COPD, End Stage Kidney Disease, tachypnea , pitting edema, ground glass opacity, decreased oxygen saturation, CRP increased, Wuhan	26
33	1	53	FEMALE	Fatigue, Fever, Cough, Hypotension, Alkalosis, Hypoxemia, Hypocapnia, Low-Voltage Electrocardiogram, ST-segment depression with T-wave inversion in lead V1 and aVR, Creatine Phosphokinase Increased, C-Reactive Protein Increased, Chloride Decreased, Potassium Increased, Hyponatremia, Lymphocytopenia, Hemoglobin Increased, Hematocrit Increased, Erythrocytes Increased, ST Elevation, Cardiac Enzymes Increased	27
33	2	53	FEMALE	fatigue, fever, cough, hypotension, tachycardia, C-reactive protein increased	27

33	3	53	FEMALE	fatigue, fever, dry cough, hypotension , alkalosis, increased creatine kinase, increased CRP, hyperkalemia, heart dysfunction	27
34	1	73	FEMALE	Diabetes Mellitus, Obesity, Chronic Kidney Insufficiency, Hypertension, Congestive Heart Failure, Cough, Fever, Dyspnea, Hypoxemia, Tachycardia, Respiratory Failure, Bilateral Pulmonary Infiltrate	28
34	2	73	FEMALE	cough, fever, dyspnea, hypoxemia, tachycardia, bilateral infiltrates CT	28
34	3	73	FEMALE	coronary artery disease, hypertension, congestive heart failure, cough, fever, shortness of breath, tachycardia , respiratory failure, bilateral infiltrates, diabetes mellitus, hypoxemia, chronic kidney disease	28
35	1	38	MALE	Fever, Lymphocytopenia, Cough, Decreased Oxygen Saturation, Dull Chest Pain, Palpitations, Dyspnea, Hypoxemia, Ground Glass Opacities (CT), consolidation, Acute Respiratory Distress Syndrome, Pulmonary Emphysema	29
35	2	38	MALE	Wuhan, Stay in COVID-19 Risk Area, fever, lymphopenia, cough, hypoxemia, chest tightness, dyspnea, Ground Glass Opacities (CT)	29
35	3	38	MALE	Covid-19 risk area , hearing loss, tinnitus, fever, decreased lymphocytes , cough, decreased oxygen saturation , chest tightness , dyspnea, hypoxemia, ground glass opacities, pulmonary lesion , consolidation	29
36	1	51	MALE	Italy, Fever, Cough, Myalgia, Malaise, Sinusitis, Sputum Production, bilateral scleral injection, Hypertension, Lymphocytopenia	30
36	2	51	MALE	fever, cough, Stay in COVID-19 Risk Area, myalgia, malaise, sputum production	30
36	3	51	MALE	fever , cough, myalgia, malaise, sinusitis, sputum production , Covid-19 risk area, hypertension	30
37	1	39	MALE	history of non-Hodgkin lymphoma, history of chronic lymphocytic leukaemia, China, Fever, Sore Throat, Productive Cough, Dyspnea, Leukocytes Increased, Lymphocytes Increased, Hemoglobin Decreased, Decreased Platelet Count, C-Reactive Protein Increased, β2-microglobulin increased, Lactate Dehydrogenase Increased, Immunoglobulins Decreased or Undetectable, Ground Glass Opacities (CT), Pleural Effusion	31
37	2	39	MALE	Stay in COVID-19 Risk Area, non-Hodgkin Lymphoma, fever, sore throat, productive cough, dyspnea, immunosupression, lymphocytosis, thrombocytopenia, C-reactive protein increased, Ground Glass Opacities (CT)	31
37	3	39	MALE	non-Hodgkin lymphoma, chronic lymphocytic leukaemia, fever, sore throat , productive cough, dyspnoea, increased CRP, lymphocytosis, thrombocytopenia, increased leukocytes , increased lactate dehydrogenase, decreased platelet count, ground glass opacities , Covid-19 risk area, China	31
38	1		MALE	Fever, Lethargy, cutaneous mottling, Respiratory Distress, Contact COVID-19 Case	32
38	2		MALE	fever, lethargy, respiratory distress	32
38	3		MALE	fever, lethargy, cutaneous mottling, respiratory distress, tachycardia, tachypnoea, contact Covid-19 patient, Iran	32
39	1	27	FEMALE	Contact COVID-19 Case, Fever, Myalgia, Cough, Lymphocytopenia, C-Reactive Protein Increased, Pneumonia	33
39	2	27	FEMALE	Contact COVID-19 Case, fever, myalgia, cough, C-reactive protein increased, CT scan abnormal	33
39	3	27	FEMALE	contact Covid-19 patient , fever, myalgia, cough, lymphocytopenia, increased CRP, pneumonia	33

40	1	26	FEMALE	Contact COVID-19 Case, Fever, Myalgia, Malaise, Cough, Sore Throat, Lymphocytopenia, C-Reactive Protein Increased, Pneumonia	33
40	2	26	FEMALE	Contact COVID-19 Case, fever, myalgia, malaise, cough, sore throat, lymphopenia, C-reactive protein increased, CT scan abnormal	33
40	3	26	FEMALE	contact Covid-19 patient, fever, myalgia, malaise, cough, sore throat, Lymphopenia, increased CRP, pneumonia	33
41	1	61	MALE	Fever, Dry Cough, Dyspnea, feeling very tired, Malaise, Hypertension, Tachycardia, Chest x-ray shows bilateral lung infiltrates, Ground Glass Opacities (CT), Respiratory Distress	34
41	2	61	MALE	fever, dry cough, dyspnea, fatigue, malaise, hypertension, tachycardia, ground-glass opacity on CT, respiratory distress	34
41	3	61	MALE	fever, dry cough, difficulty breathing, fatigue, malaise, bilateral lung infiltrates, ground glass opacity, respiratory distress, hypertension	34
42	1	26	FEMALE	Sore Throat, Dry Cough	34
42	2	26	FEMALE	sore throat, dry cough	34
42	3	26	FEMALE	sore throat, dry cough	34
43	1		UNKNOWN	Fever, Sore Throat, Myalgia, ground glass opacity of inferior lobe of left lung	35
43	2		UNKNOWN	fever, sore throat, myalgia, ground-glass opacity on CT	35
43	3		UNKNOWN	fever, sore throat, muscle pain, ground glass opacity	35
44	1		UNKNOWN	multiple patchy ground glass opacity in both lungs, effusion on both lungs, Fever, Dry Cough, Productive Cough, Headache, Fatigue	35
44	2		UNKNOWN	Fever, dry cough, productive cough, headache, fatigue, ground-glass opacity on CT	35
44	3		UNKNOWN	fever, dry cough, productive cough, headache, fatigue , ground glass opacity, effusion on both lungs	35
45	1		UNKNOWN	Fever, multiple patchy ground glass opacity on both lungs	35
45	2		UNKNOWN	ground-glass opacity on CT, Fever	35
45	3		UNKNOWN	fever, CT ground glass opacity	35
46	1		UNKNOWN	multiple patchy ground glass opacity on both lungs, Fever, Sore Throat, Dry Cough, Headache, Fatigue	35
46	2		UNKNOWN	Fever, Sore throat, dry cough, headache, fatigue, ground-glass opacity on CT	35
46	3		UNKNOWN	fever, sore throat, dry cough, headache, fatigue, ground glass opacity	35
47	1	32	MALE	Diarrhea, Fatigue, Cough, Dyspnea, Wuhan, foamy sputum, Tachypnea, Leukocytes Increased, Neutrophil Count Increased, Lymphocytopenia, C-Reactive Protein Increased, Elevated Sedimentation Rate, Glucose Increased, Alanine Aminotransferase Increased, Aspartate Aminotransferase	36

				Increased, Blood Urea Nitrogen Increased, procalcitonin increased, diffuse multiple patchy exudates, Ground Glass Opacities (CT), Hypercapnia, Hypoxemia, Base Deficit, HCO3- decreased, Lactate Increased, Acidosis, Thrombocytosis	
47	2	32	MALE	diarrhea, fatigue, dyspnea, Wuhan, hyperpnea, leukocytosis, neutrophil count increased, lymphocyte count increased, C-reactive protein increased, Erythrocyte sedimentation rate increased, alanine aminotransferase increased, aspartate aminotransferase increased, ground-glass opacities on CT	36
47	3	32	MALE	diarrhea, fatigue, cough, dyspnea , Wuhan, sputum production , tachypnea , increased leucocytes , increased neutrophil count , increased lymphocyte count, increased CRP, increased erythrocyte sedimentation rate , increased glucose , increased alanine aminotransferase, increased aspartate aminotransferase, increased blood urea nitrogen, increased procalcitonin, multiple patchy exudates, acidosis, decreased PO2, increased PCO2, ground glass opacities	36
48	1	48	MALE	Smoking, Diabetes Mellitus, Chronic Obstructive Pulmonary Disease, Fever, Cough, foamy sputum, Leukocytes Increased, Lymphocytopenia, Neutrophil Count Increased, Lactate Increased, Alanine Aminotransferase Increased, Bilirubin Increased, Plasma Fibrinogen Increased, Fibrin Degradation Products Increased, C-Reactive Protein Increased, Immunoglobulin G Increased, diffuse multiple patchy exudates with partial interlobar septation, Hypoxemia, Hypercapnia	36
48	2	48	MALE	smoking, Chronic obstructive pulmonary disease, fever, productive cough, chills, diabetes mellitus, patchy exudates on CT, leukocytosis, neutrophil count increased, lactate dehydrogenase increased, alanine aminotransferase increased, fibrinogen increased, C-reactive protein increased	36
48	3	48	MALE	fever, cough, shivering, sputum production , leukocytes increased , lymphocytes decreased , neutrophil count increased , lactate increased , increased Alanine aminotransferase, bilirubin increased , LDH increased, fibrinogen increased, CRP increased, Immunoglobulin G decreased , smoker, Diabetes mellitus , COPD	36
49	1	82	FEMALE	Tachypnea, Diarrhea, Contact COVID-19 Case, Loss of Appetite, Abdominal Distension, Decreased Oxygen Saturation, Coarse Rales, Alkalosis, Leukocytes Decreased, Lymphocytopenia, Neutrophil Count Decreased, Decreased Platelet Count, C-Reactive Protein Increased, Immunoglobulin G Increased, Hypoxemia, decreased HCO3-, bilateral ground glass opacities, peripheral exudative changes	36
49	2	82	FEMALE	Contact covid-19 case, abdominal pain, tachypnea, leukopenia, lymphopenia, neutropenia, ground-glass opacities on CT, thrombocytopenia	36
49	3	82	FEMALE	contact Covid-19 patient, Diarrhea, Nausea, decreased leukocytes , decreased lymphocytes , decreased Neutrophil count, Platelet count decreased, CRP increased , ground glass opacity, loss of appetite , abdominal distension, decreased oxygen saturation , tachypnea , alkalosis , decreased PaO2, HCO3- decreased	36
50	1	60	MALE	Hypertension, Nausea, Vomiting, Diarrhea, Fever, Dyspnea, Thrombocytopenia, bilateral parenchymal opacities, basal pleural effusions, Tachypnea, Hypoxemia	37
50	2	60	MALE	Italy, hypertension, nausea, vomiting, diarrhea, fever, dyspnea, thrombocytopenia, bilateral parenchymal opacities on X-ray	37
50	3	60	MALE	Italy , hypertension, Nausea, vomiting, diarrhoea, fever, dyspnoea , thrombocytopenia, bilateral parenchymal opacities, pleural effusions	37

Source	URL
1	http://balkanmedicaljournal.org/uploads/pdf/pdf_BMJ_2192.pdf
2	https://jkms.org/DOIx.php?id=10.3346/jkms.2020.35.e124
3	https://pubs.rsna.org/doi/10.1148/radiol.2020200236?url_ver=Z39.88-2003&rfr_id=ori:rid:crossref.org&rfr_dat=cr_pub%3dpubmed
4	https://pubs.rsna.org/doi/10.1148/radiol.2020200257?url_ver=Z39.88-2003&rfr_id=ori:rid:crossref.org&rfr_dat=cr_pub%3dpubmed
5	https://pubs.rsna.org/doi/10.1148/radiol.2020200280?url_ver=Z39.88-2003&rfr_id=ori:rid:crossref.org&rfr_dat=cr_pub%3dpubmed
6	https://pubs.rsna.org/doi/10.1148/radiol.2020200269?url_ver=Z39.88-2003&rfr_id=ori:rid:crossref.org&rfr_dat=cr_pub%3dpubmed
7	https://www.jstage.jst.go.jp/article/bst/14/1/14_2020.01030/_pdf/-char/en
8	https://www.ncbi.nlm.nih.gov/pmc/articles/PMC7029452/#!po=32.1429
9	https://www.ncbi.nlm.nih.gov/pmc/articles/PMC7029452/#!po=17.8571
10	https://www.ncbi.nlm.nih.gov/pmc/articles/PMC7036338/
11	https://www.thelancet.com/journals/lanres/article/PIIS2213-2600(20)30076-X/fulltext
12	https://www.ijidonline.com/article/S1201-9712(20)30122-3/pdf
13	https://www.ncbi.nlm.nih.gov/pubmed/32161941
14	https://onlinelibrary.wiley.com/doi/epdf/10.1111/ajt.15869
15	https://www.ncbi.nlm.nih.gov/pubmed/32100486
16	https://www.ncbi.nlm.nih.gov/pmc/articles/PMC7039714/
17	https://www.ncbi.nlm.nih.gov/pubmed/32188528
18	https://www.ncbi.nlm.nih.gov/pmc/articles/PMC7036342/
19	https://www.ncbi.nlm.nih.gov/pmc/articles/PMC7025910/
20	https://www.ncbi.nlm.nih.gov/pmc/articles/PMC7033720/
21	https://www.ncbi.nlm.nih.gov/pmc/articles/PMC7033720/#!po=31.8182
22	https://annals.org/aim/fullarticle/2763329/covid-19-risk-health-care-workers-case-report
23	https://www.nature.com/articles/s41591-020-0819-2
24	https://link.springer.com/article/10.1007%2Fs11604-020-00945-1
25	http://rimed.org/rimedicaljournal/2020/04/2020-04-50-case-tape.pdf
26	https://www.karger.com/Article/FullText/507261
27	https://jamanetwork.com/journals/jamacardiology/fullarticle/2763843
28	https://www.cdc.gov/mmwr/volumes/69/wr/mm6912e1.htm?s_cid=mm6912e1_w
29	https://www.kjronline.org/DOIx.php?id=10.3348/kjr.2020.0180
30	https://www.journalofinfection.com/article/S0163-4453(20)30148-1/fulltext
31	https://www.thelancet.com/journals/lanhae/article/PIIS2352-3026(20)30074-0/fulltext
32	https://www.ncbi.nlm.nih.gov/pubmed/32233816
33	https://www.thelancet.com/journals/lancet/article/PIIS0140-6736(20)30360-3/fulltext
34	https://bestpractice.bmj.com/topics/en-gb/3000168/case-history

35	https://www.cambridge.org/core/services/aop-cambridge-core/content/view/BBE978EC4E1C2664DE599BA639088BF7/S193578932000066Xa.pdf/case_report_on_early_diagnosis_of_covid19.pdf
36	https://www.sciencedirect.com/science/article/pii/S0929664620301443?via%3Dihub
37	https://smw.ch/article/doi/smw.2020.20246
