# Supplementary material for: Diagnostic Accuracy of Web-Based COVID-19 Symptom Checkers: Comparison Study
Source: J Med Internet Res. 2020 Oct 6;22(10):e21299. doi: 10.2196/21299 (PMC7541039; doi:10.2196/21299)
Supplement: Multimedia Appendix 4 [file jmir_v22i10e21299_app4.pdf]

Multimedia Appendix 4. List of COVID-19 symptoms according to the WHO

Fever	Fatigue	Cough	Sneezing	Malaise
Rhinorrhea	Sore throat	Diarrhea	Headache	Dyspnea
