# Supplementary material for: Diagnostic Accuracy of Web-Based COVID-19 Symptom Checkers: Comparison Study
Source: J Med Internet Res. 2020 Oct 6;22(10):e21299. doi: 10.2196/21299 (PMC7541039; doi:10.2196/21299)
Supplement: Multimedia Appendix 5 [file jmir_v22i10e21299_app5.pdf]

Multimedia Appendix 5. Mapping between symptom checkers output texts and risk levels. All mappings were independently done by two different persons and conflicts resolved by a third person’s opinion.

Ada	Your symptoms **are probably caused by other diseases** that are more common than COVID-19	Low
	It's **unlikely** that you're experiencing symptoms of COVID-19	Low
	Based on what you've reported, you should take steps to monitor your health and practice social distancing. You have **none of the typical symptoms of COVID-19.** However, in the last 14 days, you've been in contact with a confirmed or probable COVID-19 case. Also, you're in one of the groups at risk of developing a more serious form of the disease.	Medium
	The symptoms you have reported are **present in some cases** of COVID-19. You haven't reported exposure to cases of COVID-19. However, you are in one of the groups at risk of developing a more serious form of the disease.	Medium
	You have **symptoms typical of COVID-19**, and you're in one of the groups at risk of having a more serious form of the disease.	Medium
	It's **possible that COVID-19** is causing your symptoms	Medium
	There is a **high risk** that COVID-19 is causing your symptoms	High
Apple	You Should Practice Social Distancing	Low
	You Should Self-Isolate	Medium
	Contact Your Healthcare Provider	High
Babylon	It's **unlikely** that you are experiencing symptoms of coronavirus (COVID-19). But if you develop a new or continuous cough, fever or difficulty breathing, start a new symptom check.	Low
	The symptoms you mentioned sound worrying and **could be caused** by coronavirus (COVID-19). Call 999 for an ambulance immediately and let them know you may have symptoms of coronavirus.	Medium
CDC	Sorry you're feeling ill. Stay at home and monitor your symptoms. Call your provider if you get worse (No COVID-19 risk)	Low
	Stay home and take care of yourself. Call your provider if you get worse. You have one or more symptom that **may be related** to COVID-19	Medium
Cleveland Clinic	You’re at **low risk** for COVID-19.	Low
	You’re at **medium risk** for COVID-19.	Medium
	You’re at **high risk** for COVID-19.	High
Docyet	"Es besteht kein Grund zur Sorge! Basierend auf Ihren Angaben ist es **ziemlich unwahrscheinlich**, dass Sie sich mit dem Coronavirus infiziert haben. Sie zeigen keine Symptome von COVID-19.	Low
	Aktuell besteht kein wesentlicher Grund zur Sorge. Basierend auf Ihren Angaben ist es **ziemlich unwahrscheinlich**, dass Sie sich mit dem Coronavirus infiziert haben.	Low
	Aktuell besteht kein wesentlicher Grund zur Sorge. Basierend auf Ihren Angaben ist es **relativ unwahrscheinlich**, dass Sie sich mit dem Coronavirus infiziert haben. Sie schildern jedoch einige typische Symptome eines Atemwegsinfekts (z.B. Grippe, Bronchitis) oder einer Erkältung.	Low

	Sie sollten dringend ärztliche Hilfe aufsuchen. Sie haben typische Symptome einer schweren Grippe oder Lungenentzündung geschildert. Auf Basis Ihrer Angaben ist es jedoch **nicht sehr wahrscheinlich**, dass Sie sich mit dem Coronavirus infiziert haben.	Low
	Sie zeigen deutliche grippeähnliche Symptome und waren potentiell in Kontakt mit am Coronavirus infizierten Personen. Es ist **daher möglich,** dass auch Sie sich angesteckt haben.	Medium
	Sie zeigen erste grippeähnliche Symptome und waren potentiell in Kontakt mit am Coronavirus infizierten Personen. Es ist **daher möglich**, dass auch Sie sich angesteckt haben.	Medium
	"Bisher zeigen Sie keine auffälligen Symptome. Sie sind jedoch in Kontakt mit am Coronavirus infizierten Personen gekommen. Es ist **daher möglich**, dass auch Sie sich angesteckt haben, auf Grund der zweiwöchigen Ausbruchszeit jedoch noch keine Symptome zeigen.	Medium
	Ihre Symptome deuten auf eine schwere Grippe bzw. Lungenentzündung hin. Außerdem waren Sie potentiell in Kontakt mit am Coronavirus infizierten Personen. Es ist **möglich**, dass auch Sie sich angesteckt haben.	Medium
	"Sie zeigen deutliche grippeähnliche Symptome sowie erste Anzeichen einer Lungenentzündung. Außerdem waren Sie potentiell in Kontakt mit am Coronavirus infizierten Personen. Es ist **daher möglich**, dass auch Sie sich angesteckt haben.	Medium
Providence	You do not report exposure to coronavirus (COVID-19) and do not have symptoms. You don't report any symptoms of coronavirus infection, so your risk is **low**. You can use this tool again or call your provider if anything changes, but in the meantime:	Low
	You **might be** infected with coronavirus (COVID-19). Please do one of the following: Call 911 for a life-threatening emergency. Schedule an evaluation with your primary care physician. Speak with a provider at Express Care Virtual.	Medium
	Please do one of the following: Call 911 for a life-threatening emergency. Schedule an evaluation with your primary care physician. Speak with a provider at Express Care Virtual.	High
Symptoma	**High risk** for COVID-19	High
	**Medium risk** for COVID-19	Medium
	**Low risk** for COVID-19	Low
Infermedica	Your symptoms **do not suggest** that you have COVID-19. Continue following the common measures and government directives to avoid contracting COVID-19. Remember that your symptoms may also result from other diseases and may require medical consultation - this interview targets the COVID-19 infection. If your symptoms seem severe and you are worried, contact your doctor or local health authorities.	Low
	Your symptoms are worrisome and **may be relate**d to COVID-19. Call your local COVID-19-related healthcare number. Depending on the country, this may be a telephone line issued by the Ministry of Health or Health Department.	Medium
Your.MD	**There's nothing at present to suggest** that you have coronavirus (COVID-19). Please practice physical/social distancing.	Low
	You **may** have coronavirus (COVID-19).	Medium
	You **may** still be at risk of coronavirus (COVID-19).	Medium
	You're **highly likely** to have coronavirus (COVID-19).	High
	Call an ambulance immediately. Please tell them you have symptoms that may be caused by coronavirus (COVID-19).	High
