# Supplementary material for: Diagnostic Accuracy of Web-Based COVID-19 Symptom Checkers: Comparison Study
Source: J Med Internet Res. 2020 Oct 6;22(10):e21299. doi: 10.2196/21299 (PMC7541039; doi:10.2196/21299)
Supplement: Multimedia Appendix 6 [file jmir_v22i10e21299_app6.pdf]

Multimedia Appendix 6. Full table of sensitivity, specificity, accuracy, F1 score and MCC for all symptom checkers (COVID-19 positive defined by “high risk” for non binary symptom checkers)

Symptom checker	sensitivity	specificity	accuracy	F1 score	MCC
Ada	0.14	1.00	0.57	0.24	0.27
Apple	0.22	0.69	0.46	0.29	-0.10
Babylon	0.90	0.33	0.62	0.70	0.29
CDC	0.94	0.29	0.61	0.71	0.30
Cleveland Clinic	0.32	0.74	0.53	0.40	0.07
Docyet	0.16	1.00	0.58	0.27	0.29
Infermedica	0.78	0.83	0.80	0.80	0.61
Providence	0.32	0.72	0.52	0.40	0.05
SF-COS	0.90	0.66	0.78	0.80	0.58
SF-DIST	0.52	0.70	0.61	0.57	0.22
Symptoma	0.88	0.97	0.93	0.92	0.85
Your.MD	0.14	1.00	0.57	0.24	0.27
