# Supplementary material for: Diagnostic Accuracy of Web-Based COVID-19 Symptom Checkers: Comparison Study
Source: J Med Internet Res. 2020 Oct 6;22(10):e21299. doi: 10.2196/21299 (PMC7541039; doi:10.2196/21299)
Supplement: Multimedia Appendix 8 [file jmir_v22i10e21299_app8.pdf]

Multimedia Appendix 8. Constraining symptoms for Symptoma

As Symptoma exhibits the best combination of sensitivity and specificity, we focused our analysis on Symptoma's performance. Symptoma allows free-text input of one's symptoms and thereby a more precise representation of the clinical test cases. The other symptom checkers do not allow free text input which limits the number of possible symptoms considerably (Figure A). In order to investigate how Symptoma would perform if constrained, we performed pairwise comparisons where Symptoma is only allowed to use the symptoms of another symptom checker. In this setup, Symptoma is massively disadvantaged as it can not use its full abilities. For example, in the pairwise comparison with "Your.MD", Symptoma considers only "fever", "dry cough", "shortness of breath", and "contact with a confirmed COVID-19 case" for the classification of cases. The results of this analysis are summarised in Figure B, the sensitivity and specificity scatter plots are provided in the Multimedia Appendix 9 and detailed numerics in Multimedia Appendix 10 and Multimedia Appendix 11.

Under these constraints and when COVID-19 positive is defined by high risk only, Symptoma still significantly outperforms Apple and Cleveland Clinic, while performing statistically similar to six of the remaining symptom checkers (upper panel of Figure B). When COVID-19-positive is defined by high and medium risk (lower panel of Figure B), Symptoma's constrained performance is similar to seven of the other checkers, while outperforming Ada and Docyet. For Apple, Babylon, CDC, Cleveland Clinic, Providence and "Your.MD" the performance is about the same. When Symptoma is allowed to use all symptoms of the case descriptions, it clearly outperforms all other checkers (dashed blue line in Figure B). This suggests that performance is directly related to the number of symptom's any given checker considers as input, and as such, free-text input (non-constrained) will normally lead to a higher likelihood of correct diagnosis.

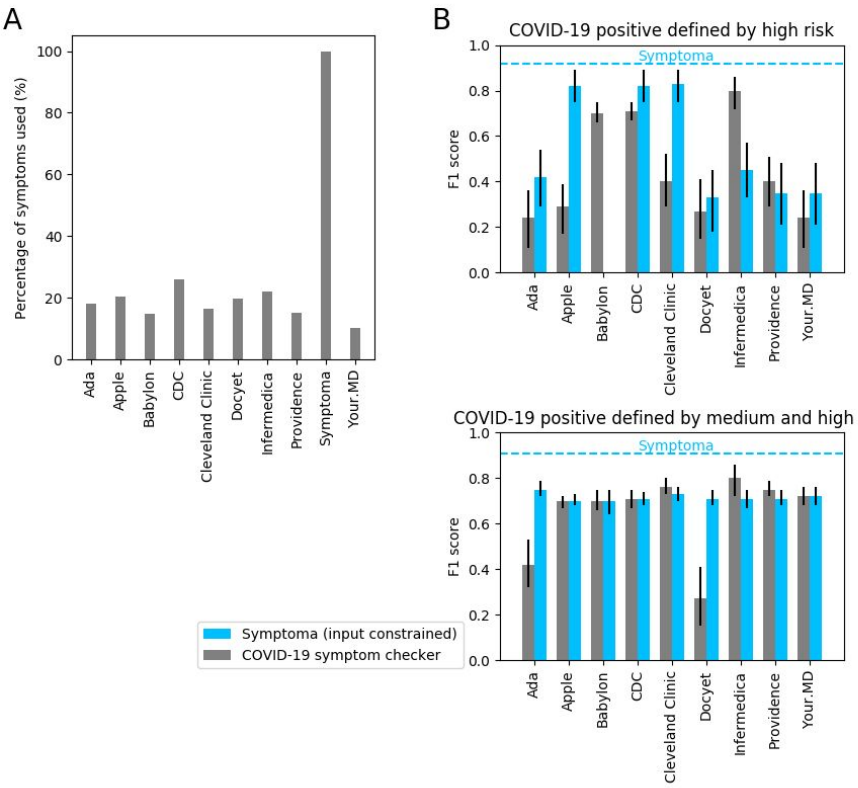

Figure. (A) Percentage of symptoms used for case classifications by each symptom checker relative to the total number of symptoms contained in all cases. (B) Symptoma input-constrained evaluation: Pairwise comparison between all symptom checkers and Symptoma based on the F_1 score if only the subset of symptoms used by one checker is also used for Symptoma. The same analysis based on the MCC is shown in the Multimedia Appendix 12. Please note that using only Babylon's symptom inputs all cases are either classified medium or low risk by Symptoma and therefore there is no bar in the upper panel for Babylon's Symptoma.
