# Supplementary material for: Diagnostic Accuracy of Web-Based COVID-19 Symptom Checkers: Comparison Study
Source: J Med Internet Res. 2020 Oct 6;22(10):e21299. doi: 10.2196/21299 (PMC7541039; doi:10.2196/21299)
Supplement: Multimedia Appendix 9 [file jmir_v22i10e21299_app9.pdf]

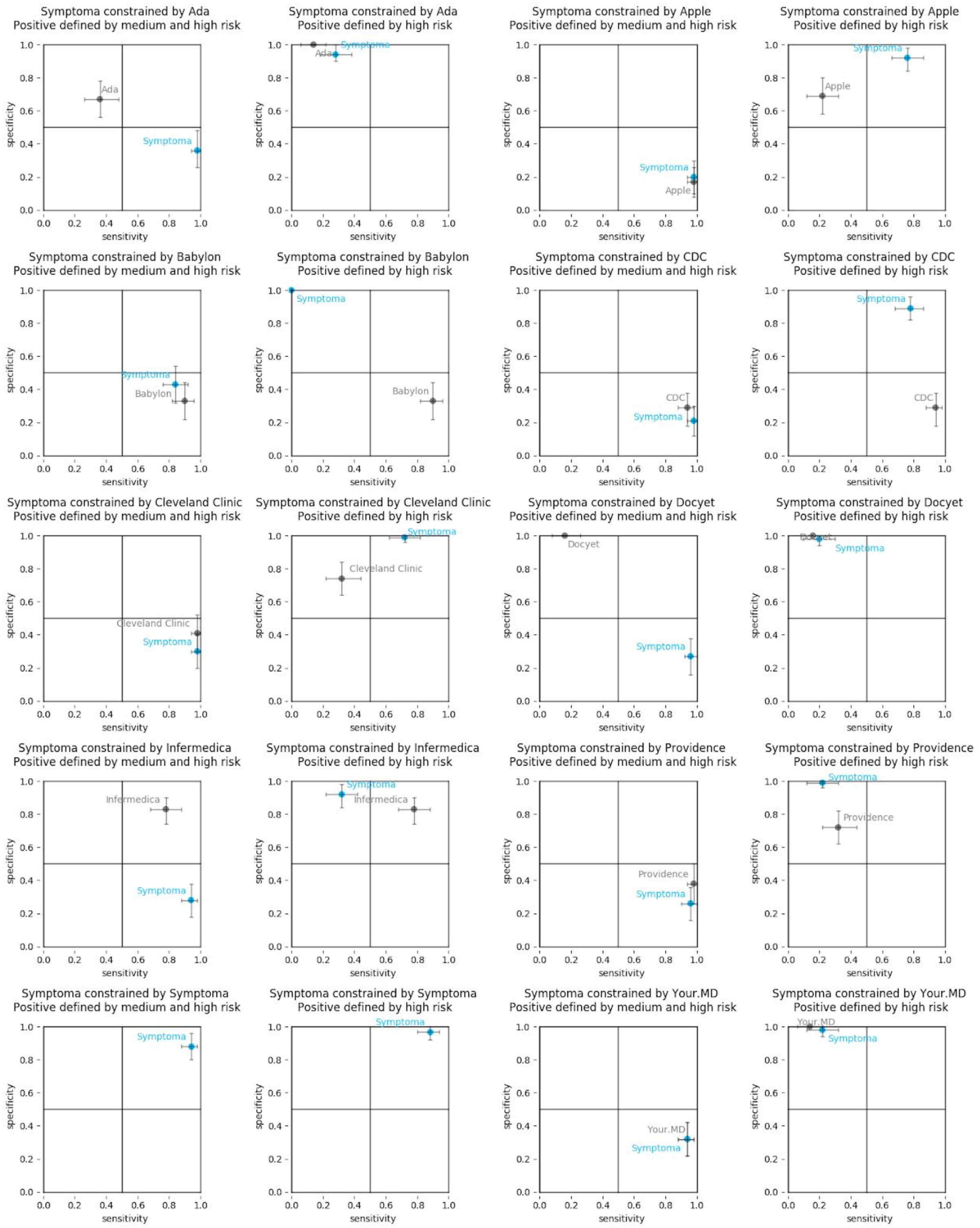

Multimedia Appendix 9. Sensitivity vs specificity for all symptom checkers and Symptoma input constraint respectively by each symptom checker
