# Supplementary material for: Diagnostic Accuracy of Web-Based COVID-19 Symptom Checkers: Comparison Study
Source: J Med Internet Res. 2020 Oct 6;22(10):e21299. doi: 10.2196/21299 (PMC7541039; doi:10.2196/21299)
Supplement: Multimedia Appendix 10 [file jmir_v22i10e21299_app10.pdf]

Multimedia Appendix 10. Full table of sensitivity, specificity, accuracy, F1 score and MCC for Symptoma constrained by each symptom checker (COVID-19 positive defined by “high risk” for non binary symptom checkers)

**Symptoma constrained by**	**sensitivity**	**specificity**	**accuracy**	**F1 score**	**MCC**
Ada	0.28	0.94	0.61	0.42	0.30
Apple	0.76	0.92	0.84	0.82	0.69
Babylon	0.00	1.00	0.50	0.00	n/a
CDC	0.78	0.89	0.83	0.82	0.68
Cleveland Clinic	0.72	0.99	0.85	0.83	0.73
Docyet	0.20	0.98	0.59	0.33	0.29
Infermedica	0.32	0.92	0.62	0.45	0.30
Providence	0.22	0.99	0.60	0.35	0.32
Your.MD	0.22	0.98	0.60	0.35	0.31
