# Supplementary material for: Diagnostic Accuracy of Web-Based COVID-19 Symptom Checkers: Comparison Study
Source: J Med Internet Res. 2020 Oct 6;22(10):e21299. doi: 10.2196/21299 (PMC7541039; doi:10.2196/21299)
Supplement: Multimedia Appendix 11 [file jmir_v22i10e21299_app11.pdf]

Multimedia Appendix 11. Full table of sensitivity, specificity, accuracy, F1 score and MCC for Symptoma constrained by each symptom checker (COVID-19 positive defined by “medium risk” or “high risk” for non binary symptom checkers)

Symptoma constrained by	sensitivity	specificity	accuracy	F1 score	MCC
Ada	0.98	0.36	0.67	0.75	0.44
Apple	0.98	0.20	0.59	0.70	0.28
Babylon	0.84	0.43	0.63	0.70	0.30
CDC	0.98	0.21	0.59	0.71	0.29
Cleveland Clinic	0.98	0.30	0.64	0.73	0.38
Docyet	0.96	0.27	0.62	0.71	0.32
Infermedica	0.94	0.28	0.61	0.71	0.29
Providence	0.96	0.26	0.61	0.71	0.31
Your.MD	0.94	0.32	0.63	0.72	0.33
