# Supplementary material for: Diagnostic Accuracy of Web-Based COVID-19 Symptom Checkers: Comparison Study
Source: J Med Internet Res. 2020 Oct 6;22(10):e21299. doi: 10.2196/21299 (PMC7541039; doi:10.2196/21299)
Supplement: Multimedia Appendix 12 [file jmir_v22i10e21299_app12.pdf]

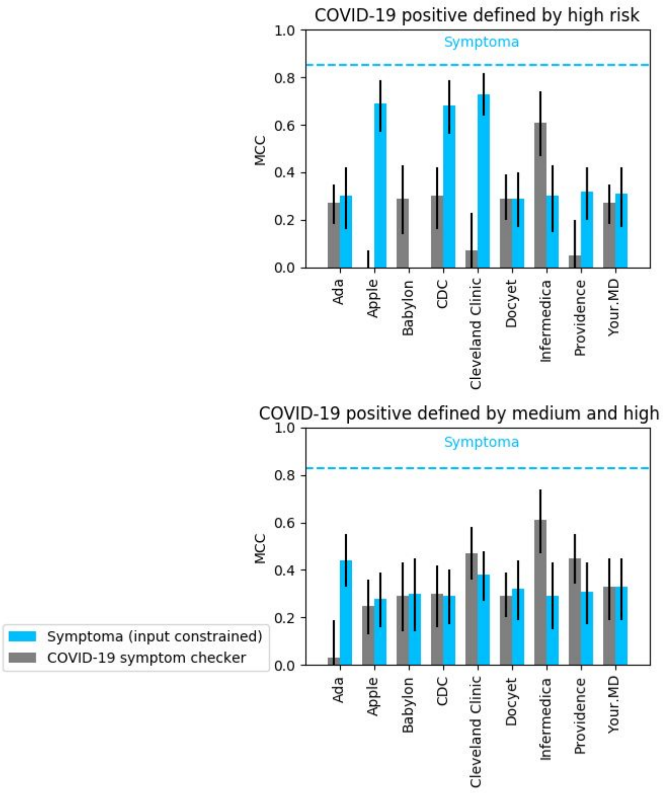

Multimedia Appendix 12. Pairwise comparison between all symptom checkers and Symptoma based on the MCC if only the subset of symptoms used by one checker is also used for Symptoma.
